# Seroepidemiological Study of Toxocariasis among Volunteers Animal Husbandry Workers and Veterinary in Southern Anatolia in Turkey in 2014

**Published:** 2015

**Authors:** Hamdi SOZEN, Burak E CITIL, Selmin CAYLAK, Aysegul A GOKMEN, Selçuk KAYA, Mustafa DEMIRCI, Metin KORKMAZ, Cem SAHIN, Ismail KIRLI

**Affiliations:** 1 *Dept. of Infectious Disease, School of Medicine, Mugla Sitki Kocman University, Mugla, Turkey*; 2 *Dept. of Microbiology, School of Medicine, Mugla Sitki Kocman University, Mugla, Turkey*; 3 *Dept. of Microbiology, School of Medicine, Katip Celebi University, Izmir, Turkey*; 4 *Dept. of Parasitology, School of Medicine, Ege University, Izmir, Turkey*; 5 *Dept. of Internal Medicine, School of Medicine, Mugla Sitki Kocman University, Mugla, Turkey*

**Keywords:** *Toxocara*, Seroprevalence, Veterinary, Animal husbandry workers, Turkey

## Abstract

***Background:*** Human toxocariasis is a parasitic infection caused by the larvae of *Toxocara* *canis*. We examine the *Toxocara* seroprevalance in veterinarians and animal husbandry workers living in the Mugla Province, Turkey to evaluate better the risk factors for *Toxocara* exposure.

**Methods: **In 2014, 376 volunteers participated in the study in 2014. All blood specimens were tested using a commercial enzyme immunoassay kit and ELISA positive samples were confirmed by Western Blot (WB) method.

**Results: **The seroprevalence of *Toxocara*, as determined by WB, was 8%. A statistically significant correlation was evident between patient age and *Toxocara* positivity among animal husbandry workers (*P* = 0.029). A strong association was also evident between sex and seropositivity in the animal husbandry group (*P*=0.024). Veterinarians working in pet clinics did in fact exhibit higher *Toxocara* seropositivities relative to those of other groups (*P* = 0.029). A statistically significant difference was detected between the rural geographic areas surveyed (*P* = 0.04).

**Conclusion: **In Mugla Province, seroprevalence of *Toxocara* is lower than other regions. Despite the low seroprevalence observed, especially in high risk professions toxocariasis remains an important medical concern within the region.

## Introduction

Human toxocariasis is a parasitic infection caused by the larvae of *Toxocara canis* and *Toxocara cati* (1, 2). Transmission to humans usually occurs because of direct contact with *Toxocara* eggs present in soil contaminated with the faeces of infected cats and dogs (3). Although most human infections are asymptomatic, clinical syndromes include visceral larva migrans and ocular larva migrans (4). Significant differences in disease manifestations are also evident between children and adults, with children more likely to exhibit symptoms of covert toxocariasis, compared with common toxocariasis in adults (5-6).

Clinical diagnosis of *Toxocara* infections can be achieved using a variety of methods. Unlike many parasitic infections, stool examination alone is rarely sufficient to diagnose an infection (7); additional confirmation is necessary using molecular methods, such as ELISA or Western blotting. While the latter technique can definitively confirm an infection, the high sensitivity and specificity of ELISA-based methods using antigens secreted by the nematode have made ELISA the standard method of detection in most clinical laboratories (8).

As with most parasitic infections, seroprevalence tends to be higher in developing countries, although *Toxocara* is also common in many first-world countries. Risk factors for toxocariasis include dog ownership, children with pica, outdoor parks in urban and suburban settings, urban environments, and the presence of semi-wild dog and cat populations, as evident in many tropical and subtropical regions. However, in regions such as Mugla, Turkey, dogs and cats are more commonly found in rural areas, leading to a disproportionate number of toxocariasis infections among rural, rather than urban, populations. In regions such as this, children, veterinarians, and those engaged in animal husbandry are considered to be at greater risk (9). 

Here, we examined the *Toxocara* seroprevalance in veterinarians and animal husbandry workers living in the province of Mugla, Turkey, to evaluate better risk factors for *Toxocara* exposure.

## Materials and Methods

This research was approved by the Ethics Committee of Katip Celebi University Faculty of Medicine, Izmir, Turkey. This cross-sectional study was conducted in the province of Mugla, Turkey, between 01/11/2013 and 01/08/2014. 

***Study Population***

The study population consisted of volunteers selected at random from persons living in different geographical regions of Mugla Province. Among animal husbandry workers, 251 volunteers were selected from six different geographic regions of Mugla Province: Yerkesik, Sarayyani, Toparlar, Bencik, Kafaca, and Yilanli. In terms of veterinarians and veterinary technicians, 125 volunteers were selected from 7 different geographic regions: Mentese, Yatagan, Marmaris, Milas, Koycegiz, Fethiye, and Bodrum.

***Sample Collection***

Volunteers were asked to complete a questionnaire detailing relevant socioeconomic and occupational risk factors, and 10 mL amounts of blood were collected. Blood samples were centrifuged at 2,500 rpm for 15 min, and the sera stored at -80 ^o^C until analysis.

***ELISA***

All specimens were tested using a commercial enzyme immunoassay kit (DRG *Toxocara canis* EIA-3518, Lot No:1519, DRG International, Springfield, NJ, USA) in accordance with the manufacturer’s instructions. Briefly, all sera were diluted 1:64 in dilution buffer and transferred to test wells. Antigen-antibody complexes were detected using an anti-IgG enzyme conjugate, and visualised with a chromogen (tetra-methylbenzidine; TMB). Colour intensity was measured using a Biotek ELx800 (Biotek, Winooski, VT, USA), with absorbances being read at 450 and 630 nm. Samples yielding absorbance values ≥ 0.3 OD units were considered positive.

***Western Blots***

Our study WB test was held in Ege University Faculty of Medicine Parasitology Laboratory. Western blot (WB) assay was performed using excretory/secretory antigens (ES) of *T. canis* second stage larvae (10). ES antigens electrophoretically separated by Mini-Protean III electrophoresis cells (Bio-Rad) with 10% running sodium dodecyl sulfate polyacrylamide-gel (SDS-PAGE), 4% stacking gel, using the buffer system described by Laemmli (11), and transferred to nitrocellulose sheets (Protran BA 83, Schleicher&Schuell) by semi-dry electroblotting (EBU-4000, C.B.S. Scientific). Blots were blocked with 0.5% casein in phosphate-buffered saline, pH: 7,2 (PBS-CB), washed with PBS, and then cut into 2-mm strips. Strips were incubated with 1:50 dilutions of patient sera in PBS-CB for 90 min, washed with PBS three times, incubated with alkaline phosphatase-conjugated goat anti-human immunoglobulin G (Sigma), diluted 1:5,000 in PBS-CB for 90 min, and washed three times with PBS. All incubations were performed at room temperature on a rotatory shaker. Antibody reactivity was visualized with 5-bromo-4-chloro-3-indolylphosphate and toluidinium-nitroblue tetrazolium substrate. For accuracy, strips were fixed in the original position as they were cut from the membrane. The results were considered as positive, when the samples react to two or more low-molecular-weight bands (LMWB 30-45 kDa) (8).

***Statistical Analyses***

Data were analysed using SPSS version 20.0 software for Windows (SPSS Inc., Chicago, IL, USA). The distributions of continuous variables were evaluated using the Kolmogorov-Smirnov test. Normally distributed variables were log-transformed as required. Non-normally distributed numerical variables are expressed as medians, and compared using the Mann-Whitney U test; the chi-squared test was used to compare categorical variables. *P* values < 0.05 were considered statistically significant.

## Results

A total of 376 volunteers participated in the study, including 125 (33.2%) veterinarians and veterinary technicians, and 251 (66.8%) animal husbandry workers. The seroprevalence of *Toxocara* was 8.5% across all study participants, as determined by ELISA, with a higher degree of seroprevalence evident in animal husbandry workers relative to veterinarians and veterinary technicians (9.2% vs. 7.2%, respectively). 

**Fig. 1:**
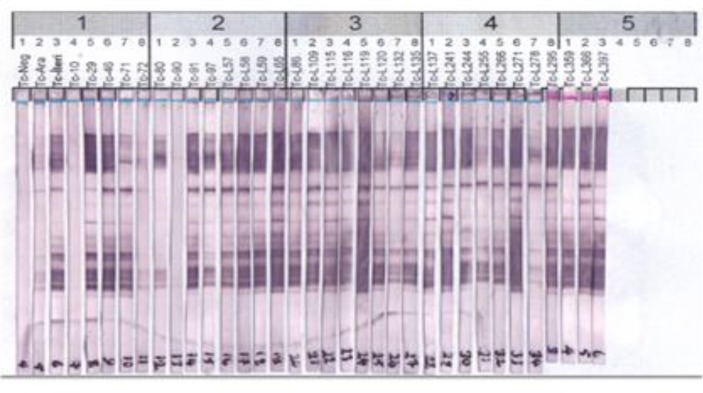
*Toxocara* ELISA test (+) detected in patients with Westren blot test image

Next, samples identified as potential positives by ELISA were validated by WB. Overall, 30 of 32 samples (93.75%) were also positive by WB, indicating that both assays afforded high specificities (Fig. 1). Both samples identified as positive by ELISA, but negative by WB, were from either veterinarians or veterinary technicians. Consequently, the overall seroprevalence of *Toxocara*, as determined by WB, was 8%. Upon such analysis, the seroprevalence in animal husbandry workers remained steady at 9.2%, whereas that in veterinarians and veterinary technicians fell to 5.6%; this between-occupation difference was not statistically significant (*P* = 0.230).

The median age of all volunteers was 45 years (range 20-79 years), and 210 males (55.9%) and 166 females (44.1%) were tested. The median age was slightly higher among animal husbandry workers, at 49 years (range: 20-79 years), with a disproportionately high female to male ratio (143:108), relative to the study population as a whole. 

**Table 1:** The volunteers who participated in the study engaged, WB *Toxocara* seropositivity rates

**Parameters**		● **AH-(n)**	● **AH-Toxocara Seropositivity**	● **AH-Statistic**	◘ **VET-(n)**	◘ **VET-Toxocara Seropositivity**	◘ **VET-** **Statistic**	**Total (n)**	**Total Toxocara Seropositivity**	**Total Statistic**
			**n (%)**			**n (%)**			**n (%)**	
Sex	Female	143	8 (5.6)	*P*=0.028*	23	1(4.3)	*P*=0.772	166	9 (5.4)	*P*=0.056
	Male	108	15 (13.9)		102	6(5.9)		210	21(10)	
Ages	20-39	74	8 (10.8)	*P*=0.064	75	5(6.7)	*P*=0.525	149	13 (8.7)	*P*=0.029*
	40-59	109	5 (4.6)		50	2(4.0)		159	7 (4.4)	
	60-more	68	10 (14.7)		0	0(0)		68	10 (14.7)	
Dog/cat ownership	Yes	178	19 (10.7)	*P*=0.195	47	2(4.3)	*P*=0.612	225	21 (9.3)	*P*=0.211
	No	73	4 (5.5)		78	5(7.8)		151	9 (5.9)	
Total		251	23 (9.2)		125	7(5.6)		376	30 (8)	

● AH: Animal husbandry workers

◘ VET: Veterinarians

P : *P* value

* *P*: *P*-value of less than 0.05 was considered significant.

In veterinarians and veterinary technicians, the median age was much lower at 37 years (range 26-59 years); we tested 102 males (81.6%) and 23 females (18.4%). All veterinarians and veterinary technicians lived in urban areas, while animal husbandry workers were exclusively rural.

A statistically significant correlation was evident between patient age and *Toxocara* positivity (by WB) among animal husbandry workers (*P* = 0.029; Table 1); similar associations were not seen in either the veterinarian or veterinary technician groups or the study population as a whole (*P* = 0.064 and *P* = 0.525, respectively). A strong association was also evident between sex and seropositivity in the animal husbandry group (*P* = 0.024); no such association was evident among veterinarians or veterinary technicians (*P* = 0.772). Taken as a whole, the relationship between sex and seropositivity was not statistically significant (*P* = 0.056); however, the relative strength of the association may be potentially meaningful (Ta ble 1). No difference was seen when living region and the extent of seropositivity were compared (*P* = 0.218).

Of all study participants, 225 of 376 (59.8%) reported having a dog or cat at home; a larger percentage of animal husbandry workers (70.9%) lived with dogs and cats, compared to veterinarians and veterinary technicians (37.6%). Despite the relationship between *Toxocara* seropositivity and a dog or cat at home, no statistically significant association was evident among any of the three groups (*P*= 0.211, *P* = 0.195, and *P* = 0.612, for all participants, animal husbandry workers, and veterinarians and veterinary technicians, respectively; Table 1). 

A statistically significant difference was detected between the rural geographic areas surveyed (*P* = 0.04). *Toxocara* seropositivity was found higher in a residential area with low socioeconomic level. The most common sites of *Toxocara* seropositivity were Toparlar (17%), Sarayyani (13.2%), and Kafaca (12.1%); in contrast, *Toxocara* seropositivity has never been described in either Yerkesik or Bencik (Table 2). In veterinarian and veterinary technician, no such association was evident between any of the urban locations described (*P* = 0.505). However, the comparison was rather complicated among veterinarians and veterinary technicians, of whom 23.2% worked in pet clinics, while the remaining 76.8% worked primarily with livestock such as sheep, goats, and cows. Veterinarians and veterinary technicians working in pet clinics did in fact exhibit higher *Toxocara* seropositivities relative to those of other groups (*P* = 0.029) (Table 3).

**Table 2:** Engaged in animal husbandry, *Toxocara* positivity rates

*Regions	**Number of ** **seropositive serums**	**Number of ** **seronegative serums**	**(%)**
Yerkesik	0	8	0.0
Sarayyani	5	33	13.2
Toparlar	8	39	17.0
Bencik	0	58	0.0
Kafaca	7	51	12.1
Yilanli	3	39	7.1
Total	23	228	9.2

Engaged in animal husbandry, between Toxocara positivity and the settlements, there is a statistically significant (*P*=0.04).

Regions: Residential areas that serum samples were collected

**Table 3:** *Toxocara* WB seropositivity rates among Veterinarians and veterinary technicians

**Working area of veterinarians**	**Number of ** **seropositive serums**	**Number of ** **seronegative serums**	**(%)**
Livestock	3	93	3.12
Pet animals	4	25	13.14
Total	7	118	5.6

Significant statistically difference was detected between Toxocara WB positivity and working area of veterinarians (*P*=0,029).

## Discussion

We observed statistically significant associations between *Toxocara* seropositivity and male animal husbandry workers, advanced age, and veterinarians working primarily with pet animals. Furthermore, among animal husbandry workers, *Toxocara* seropositivity rates were significantly higher in lower socioeconomic levels’ geographic locations, with no case of toxocariasis ever reported in some regions.

The overall *Toxocara* seroprevalence was 8%, considerably lower than that of previous studies. In work conducted in three different regions of the Turkey, *Toxocara* seroprevalence ranged from 21.4% to 12.9% (12-14). Other studies conducted in healthy individuals in China and the United States reported *Toxocara* seroprevalence rates of 13.07% and 13.9%, respectively (4, 15), thus significantly higher than described here. The studies, which have been conducted on school children in Iran; the researchers have stated different prevalence values from western and southern parts of the country 8.8% and 25.6% respectively (16, 17) .

One explanation for the lower overall seropositivity rate observed may be that volunteer populations were studied. *Toxocara* seropositivity has been found to be higher in children and the elderly (18, 19), neither of whom was included in our work. The older animal husbandry workers we surveyed were more likely to be seropositive for *Toxocara*, consistent with prior observations, although the mechanisms underlying this association remains unclear, with potential causes including increased exposure over time, weakened immunity, or deteriorating hygiene.

Male sex has been identified as an independent risk factor for *Toxocara *seropositivity (12, 20-22); however, the opposite has also been reported (13, 15). In our study, gender was not a significant risk factor among veterinarians and veterinary technicians, but was among animal husbandry workers, with seropositivity being significantly more common in men. 

The faeces of infected dogs and cats are the primary reservoir of *Toxocara* eggs. Infection is primarily caused by ingestion of embryonated eggs, with children being the most susceptible population. Not surprisingly, *Toxocara* seropositivity has been found to be highest in patients with a history of feeding domestic cats and dogs (23, 24), although this association was not found to be an independent risk factor in our study (25). The study populations described here are at particular risk for exposure to animal faeces because of their occupations, to the point at which it becomes difficult to isolate the effects of exposure to pet animals. However, a causal association between exposure and seroprevalence was evident among veterinarians working in pet clinics, with seroprevalence being significantly higher than in veterinarians working primarily with sheep and cows.

Veterinarians, farmers, and pet-shop workers are all at increased risk of toxocariasis, relative to that of other professions (4, 26). In our study, no significant difference was observed between animal husbandry and veterinary workers in terms of *Toxocara* seroprevalance. Those living in rural areas are at an increased risk of toxocariasis because of increased exposure to pets, livestock, and contaminated soil; inadequate infrastructure; lower educational levels; lower overall compliance with good personal hygiene practices; and more exposure to stray dogs and cats (27-29). Here, we divided the study volunteers into groups based on geographic location, with veterinary workers residing in urban areas and animal husbandry workers living in rural areas. Additional comparisons between rural and urban exposures and seroprevalence are therefore unnecessary.

Animal husbandry workers resided in six different settlements. The highest levels of *Toxocara* seropositivity were observed in Toparlar, Sarayyani, and Kafaca settlements. In contrast, no cases were detected in either Bencik or Yerkesik, indicating a significant distinction between locations despite widespread similarities in terms of both geography and demographics. Additional studies are necessary to understand fully this distinction; including screening pets for seropositivity to better understand the potential reservoirs for disease transmission. No apparent geographic difference was evident among veterinarians, consistent with differences between rural and urban seroprevalence.

## Conclusion

The data presented here suggest that overall seroprevalence of *Toxocara *in Mugla province is lower than that in other regions. However, local hotspots of *Toxocara *seroprevalence were evident, with strong differences noted among municipalities; expansion of screening efforts to include house pets may be warranted in some areas to identify the causes of such differences. Despite the low seroprevalence observed in this study, toxocariasis remains an important medical concern within the region. Future studies will include a more representative cross-section of the Mugla population, allowing a more detailed understanding of the prevalence and distribution of *Toxocara* throughout the region. By better understanding the reservoirs and route of transmission for *Toxocara* in this region, we will improve our ability to diagnose and prevent disease outbreaks.
